# Implementation of a methodology for determining elastic properties of lipid assemblies from molecular dynamics simulations

**DOI:** 10.1186/s12859-016-1003-z

**Published:** 2016-04-12

**Authors:** Niklaus Johner, Daniel Harries, George Khelashvili

**Affiliations:** Swiss Institute of Bioinformatics, Klingelbergstrasse 50/70, Basel, Switzerland; Institute of Chemistry and the Fritz Haber Research Center, The Hebrew University, Jerusalem, 91904 Israel; Department of Physiology and Biophysics, Weill Medical College of Cornell University, New York, NY 10065 USA

**Keywords:** Bending rigidity, Lipid tilt and splay, Helfrich theory of elasticity, Tilt modulus, Splay modulus

## Abstract

**Background:**

The importance of the material properties of membranes for diverse cellular processes is well established. Notably, the elastic properties of the membrane, which depend on its composition, can directly influence membrane reshaping and fusion processes as well as the organisation and function of membrane proteins. Determining these properties is therefore key for a mechanistic understanding of how the cell functions.

**Results:**

We have developed a method to determine the bending rigidity and tilt modulus, for lipidic assemblies of arbitrary lipid composition and shape, from molecular dynamics simulations. The method extracts the elastic moduli from the distributions of microscopic tilts and splays of the lipid components. We present here an open source implementation of the method as a set of *Python* modules using the computational framework *OpenStructure*. These modules offer diverse algorithms typically used in the calculatation the elastic moduli, including routines to align MD trajectories of complex lipidic systems, to determine the water/lipid interface, to calculate lipid tilts and splays, as well as to fit the corresponding distributions to extract the elastic properties. We detail the implementation of the method and give several examples of how to use the modules in specific cases.

**Conclusions:**

The method presented here is, to our knowledge, the only available computational approach allowing to quantify the elastic properties of lipidic assemblies of arbitrary shape and composition (including lipid mixtures). The implementation as python modules offers flexibility, which has already allowed the method to be applied to diverse lipid assembly types, ranging from bilayers in the liquid ordered and disordered phases to a study of the inverted-hexagonal phase, and with different force-fields (both all-atom and coarse grained representations). The modules are freely available through GitHub at https://github.com/njohner/ost_pymodules/ while OpenStructure can be obtained at http://www.openstructure.org.

## Background

The existing compositional heterogeniety among membranes of various cell organelles, compartments of membranes, and even between leaflets of the same lipid bilayer [1], attests to the tight cellular control over lipid species production and localization [2, 3]. Such control is neccessary because the lipid composition of a membrane determines its physical properties, which play an important role in the many functions of the cell [4–7]. Recent studies have demonstrated how lipid mixtures can affect various properties of the membrane, notably the lateral pressure profile and dipole potential [8] as well as its mechanical properties [9]. It has also been shown that lipid mixtures can present radically different properties than their constituents, e.g., the large fluctuations in bilayer thickness observed for DMPC (dimyristoylphosphocholine) / DSPC (distearoylphosphocholine) mixtures, but not for either DMPC or DSPC alone [10].

The mechanical properties of the membrane are thought to be a key determinant of how the lipid composition affects diverse physiological processes in the cell. Apart form their obvious role in processes involving membrane reshaping [11, 12], such as budding and fusion, their impact on membrane protein organisation and function has been long studied [4, 13, 14]. For example, the rate of pore formation by gramicidin depends directly on the elastic properties of the embedding lipid bilayer [15]. Other well-known examples include activation of rhodopsin and gating of meachanosensitive channels [14]. Our mechanistic understanding of these processes hence depends on knowledge of the material properties of physiological membranes in all their diversity of composition and shape.

Several methods have been developed to extract the bending rigidity of lipid bilayers from molecular dynamics (MD) simulations [16–18], in order to complement the more time-consuming and costly experimental techniques [19]. While these computational approaches have been demonstrated to predict bending rigidities for single-component bilayers in good agreement with experimental measurements, they usually require the simulation of large membrane patches (typically at least on the order of 1000 lipids) as they rely on the spectral analysis of the undulations of the bilayer. Furthermore, implementation of these computational methodologies for multi-component lipid membranes or for non-planar lipidic phases has not been reported.

Recently, we have introduced a new method to extract the tilt modulus and bending rigidity from MD simulations by following the microscopic fluctuations of lipid tilts and splays [20, 21]. The method has been demonstrated to be applicable to lipidic assemblies of arbitrary shape and composition. We showed that the method could be used to reliably calculate the bending rigidity from MD simulations of relatively small-size lipid systems (a few hundred lipids) and for a range of assemblies that include bilayers with ternary mixtures of lipids in the liquid ordered and disordered phases [21], as well as binary mixtures of lipids in the inverted-hexagonal phase [20].

## Implementation

The method was implemented as a set of python modules, which allow a flexible use of the method and its application to a range of systems going from simple bilayers to multicomponent complex lipidic phases. The implementation heavily relies on the open source computational structural biology framework OpenStructure [22, 23] for all basic operations performed on structures, trajectories and density maps. This includes all input/output (i/o) operations (reading and writing structures, trajectories and density maps), selecting parts of a structure, finding neighboring residues, applying rotations and translations to a structure, calculating density maps from atomic positions, as well as performing vectorial operations. The implementation also relies on several other freely available python modules, notably numpy, scipy [24] and matplotlib [25].

### Theoretical background

While a complete derivation of the theory behind the method can be found elsewhere [9, 20, 21, 26–28], we introduce here the main variables and expressions essential for understanding the usage and output of the different functions implemented in the python modules.

We first examine the tilt modulus. One can show [9, 20] that the probability for a lipid to be tilted by a small angle *θ* is related to the lipid tilt modulus *κ*_*t*_^*l*^ by:

1 $$ P\left(\theta \right)= Csin\theta \exp \left(-\frac{\kappa_t^l{\theta}^2}{2{k}_BT}\right) $$

where *C* is a constant, *θ* is the angle between the lipid director $\overrightarrow{n}$ and the membrane normal $\overrightarrow{N}$, and *k*_*B*_ and *T* have their usual meaning of Boltzmann’s constant and temperature, respectively. Our strategy is to calculate the distribution of tilt angles *P*(*θ*) from a well-converged MD simulation trajectory and then extract the lipid tilt modulus from a quadratic fit to the following expression [9, 20]:

2 $$ a+b{\theta}^2 = -{k}_BTln\left(\frac{P\left(\theta \right)}{sin\theta}\right) $$

where $-{k}_BTln\left(\frac{P\left(\theta \right)}{sin\theta}\right)$ is the potential of mean force (PMF) for tilting a lipid. The lipid tilt modulus then corresponds to *κ*_*t*_^*l*^ = 2*b*.

The strategy to obtain the bending rigidity is similar, except that the modulus is extracted from the distribution of lipid splays instead of tilts. Specifically, we define the lipid splay *S*_*i*_ as the covariant derivative of the vector field $\overrightarrow{n}-\overrightarrow{N}$ along one direction on the membrane interface, which can be written as

3 $$ {S}_i\left(\overrightarrow{p}\right)=\underset{h\ \to\ 0}{ \lim}\frac{n_i\left(\overrightarrow{p}+h\overrightarrow{e_i}\right)-{n}_i\left(\overrightarrow{p}\right)+{N}_i\left(\overrightarrow{p}+h\overrightarrow{e_i}\right)-{N}_i\left(\overrightarrow{p}\right)}{h} $$

where $\overrightarrow{e_i}$ is a vector tangent to the membrane and ${n}_i\left(\overrightarrow{p}\right)$ and ${N}_i\left(\overrightarrow{p}\right)$ are the components along $\overrightarrow{e_i}$ of the lipid director and membrane normal vector fields at the point $\overrightarrow{p}$ on the membrane surface. It should be noted that *S*_*i*_, referred to here as “the lipid splay”, is not the same quantity as the one commonly used to represent splay $S=\overrightarrow{\nabla}\left(\overrightarrow{n}-\overrightarrow{N}\right)$. Importantly, one can show [20] that the distribution of lipid splays, provided they are weakly correlated, follows:

4 $$ P\left({S}_i\right)=C \exp \left(-\frac{K_c{S}_i^2{A}_L}{2{k}_BT}\right) $$

with *A*_*L*_ being the area per lipid. Hence the bending rigidity *K*_*c*_ can be obtained from a quadratic fit

5 $$ a+b{S}_i^2=-{k}_BTln\left(P\left({S}_i\right)\right) $$

with − *k*_*B*_*Tln*(*P*(*S*_*i*_)) being the PMF for splaying a pair of lipids and with the bending rigidity obtained from *A*_*L*_*K*_*c*_ = 2*b*.

It should be noted that Eqs. (4) and (5) are strictly valid only at the neutral plane. While this does not influence calculations done on planar bilayers, in the case of curved membranes the value of the lipid splay may change depending on the choice of the plane at which it is calculated. In practice, the neutral and pivotal planes are usually close to each other [29]. As the pivotal plane is more convenient to determine from molecular simulations (because it is the plane where the area per lipid does not change with membrane curvature), we assume, for simplicity, that the two planes coincide. This assumption is also routinely used in most experimental determinations of membrane rigidity [29]. An example for this procedure can be found in Ref. [20], where we have shown how to determine the pivotal plane from an aligned MD trajectory of an inverted hexagonal phase. Since the procedure requires a well-aligned MD trajectory, our implementation includes a robust algorithm for aligning MD trajectories from simulations of lipid assemblies of complex geometry as detailed in refs. [20, 30] and described brielfy in the following.

### Alignment of trajectories and extraction of the normal vector field

**Fig. 1:**
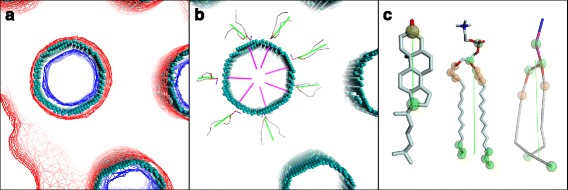
Lipid and water densities and interface. (**a**) Illustration of the determination of the lipid water interface for a DOPE (dioleoylphosphoethanolamine)/Cholesterol inverted-hexagonal phase [20]. The lipid density map is shown in red, the solvent density in blue and the interface as cyan spheres. (**b**) Same system as (a), showing the interface in cyan spheres with several normal vectors shown in magenta and DOPE lipids with their director vectors shown as in (c). (**c**) Examples of selections used as headgroup, tail, and to calculate distances between lipids. Atoms used to define the director vector (headgroup and tail) are shown as green translucent spheres, atoms used in distance calculation as orange, and atoms used for both are in khaki. Director vectors are shown as green lines. From left to right, we show an all-atom cholesterol and DPPC (dipalmitoylphosphocholine) lipid (CHARMM force-field [36]) and a coarse-grained DOPE lipid (Martini force-field [37]). For phospholipids we typically use the last three carbon atoms of each acyl chain as the tail position, whereas we use several atoms from the hydrophilic region to define the head group (for example the phosphate atom and the first carbon atom linking the two acyl chains). For cholesterol we use the carbon of the carboxyl group and the last carbon of the rigid aromatic region of the molecule as head and tail respectively. Atoms used for distance calculations should correspond to atoms lying on the pivotal plane and hence depend on the system studied, but typically the pivotal plane is situated just below the hydrophilic region of the membrane and hence, for phospholipids, the first few carbon atoms of the acyl chains can be used. The corresponding dictionaries for the examples given here are: *tail_dict={'CHL1': 'aname=C17', 'DPPC': 'aname=C214, C215, C216, C314, C315, C316', 'DOPE': 'aname=C5A, C5B'}*, *head_group_dict={'CHL1': 'aname=C3', 'DPPC': 'aname=P, C2', 'DOPE': 'aname=PO4, GL1, GL2'}* and *distance_dict={'CHL1': 'aname=C3', 'DPPC': 'aname=C22, C21, C23, C31, C32, C33', 'DOPE': 'aname=C1A, C1B'}*

We define the field of normal vectors $\overrightarrow{N}$ using the (time) averaged shape of the lipid-water interface, while tilt and splay degrees of freedom represent fluctuations around this average shape. This implies that, in the simulations of a finite size planar lipid bilayer patch with periodic boundary conditions, the normal vector field will be constant (typically $\overrightarrow{N}=\overrightarrow{e_z}$) because the membrane is flat on average. The definition of the normals becomes more complicated for curved membranes such as the ones found in complex lipidic phases like the inverted-hexagonal phase [20] (see Fig. 1a for a representation). Indeed, in such cases it is necessary to first properly align the trajectory frames prior to extracting the average lipid-water interface to calculate the field of vectors normal to the interface. The alignment can be performed by maximizing the overlap of the solvent molecules with the density map of the solvent in a reference frame while minimizing the overlap of the lipid component with that same density. Specifically, the density map is calculated as a sum of gaussians [31]

6 $$ {\rho}_W^{\mathrm{j}}\left(\overrightarrow{y}\right)={\displaystyle \sum_{i=1}^{N_W^{ref}}}C{m}_iexp\left(-D\parallel \overrightarrow{y}-\overrightarrow{x_{ij}^s}{\parallel}^2\right) $$

where $\overrightarrow{x_{ij}^W}$ is the position of the *i*^*th*^ out of the *N*_*W*_^*ref*^ solvent (water) molecules in the reference frame *j*, *m*_*i*_ is its mass, and *C* and *D* are constants that depend on the chosen resolution of the map [31]. This density map is then smoothed using a low-pass filter and then, for each frame *k*, we seek the transformation (rotation and translation) maximizing

7 $$ {\displaystyle \sum_{i=1}^{N_W}}{\rho}_W^j\left(\overrightarrow{x_{ik}^W}\right)-{\displaystyle \sum_{i=1}^{N_L}}{\rho}_W^j\left(\overrightarrow{x_{ik}^L}\right) $$

where $\overrightarrow{x_{ik}^L}$ are the position of the *N*_*L*_ lipid molecules.

The water-lipid interface is then determined from the aligned trajectory as follows. First we calculate the average density over all aligned frames for the solvent and for the lipids. Then we determine the water-lipid interface as the surface (on a 1Å mesh) where these densities are equal. This procedure is illustrated in Fig. 1a (see also Fig. S2 of ref. [30] for more details).

Normal vectors are then simply calculated for each point of the interface from a best fit plane, typically to all points of the surface within 10Å (Fig. 1b).

### Molecular definitions of tilts and splays

The director vector for a lipid is defined as a vector pointing from the tail of the lipid to its head. We typically define this vector as the one joining the center of mass of the last carbon atoms of each chain to the center of mass of several heavy atoms in the headgroup. The specific choice of the atoms in the definition depends on the level of representation used (i.e. all-atom vs coarse-grained) and the lipid species, and can be controlled by the user (see below). An example of such a definition for different lipid species can be found in Fig. 1c. We have verified [21] that the exact definition used for the director vector will have only a small impact on the calculated elastic moduli, and trends observed in the differences of moduli between different systems and conditions should be preserved.

In our implementation, we use dictionaries to define the selection of atoms representing the headgroup and tail for each lipid species. These dictionaries have one entry for each lipid species (residue name) associated with a *query string* [22, 23] for selecting the atoms corresponding to the lipid headgroup or tail for that lipid species (see examples given in Fig. 1c).

For each lipid, the tilt angle in each frame is calculated as the angle between its director vector and the normal vector of the point of the interface closest to the lipid headgroup.

Splays are calculated for lipid pairs using the finite difference eq. (3) with $\overrightarrow{n}\left(\overrightarrow{p}\right)$ the director vector of the first lipid, $\overrightarrow{n}\left(\overrightarrow{p}+h\overrightarrow{e_i}\right)$ that of the second lipid, and $\overrightarrow{N}\left(\overrightarrow{p}\right)$ and $\overrightarrow{N}\left(\overrightarrow{p}+h\overrightarrow{e_i}\right)$ the normal vectors of the points of the interface closest to the first and second lipid respectively. $\overrightarrow{e_i}$ is the unit vector pointing from the first to the second lipid and perpendicular to $\overrightarrow{N}\left(\overrightarrow{p}\right)$ and *h* is the distance between the lipids. We restrict the calculation of splays to pairs of lipids that are close together, typically using a cutoff of *h* < 10*Å*. This cutoff should be small enough to maintain the validity of the calculation of the lipid splay from a finite difference, while still sufficiently large to guarantee that the splays are independent. A cutoff of 10Å, which basically restricts the calculations to nearest lipid neighbors, is justified by the fact that the spatial correlations in lipid splay decay on length scales similar to the linear dimension of a lipid [32] (i.e. ~8Å).

The distance between lipids is measured as the distance between the centers of mass of a selection of atoms in each lipid in the pair. As for headgroups and tails, the selections used for distance calculations are defined in a dictionary containing an entry for every lipid species present in the system.

### Periodic boundary conditions

Several issues make the treatment of periodic boundaries challenging, the main one being that after aligning a trajectory, it can become impossible to store the unit cell vectors in most available file formats for trajectories, such as in *dcd* files, which assume that the first vector is along the *x direction *. Moreover, several of the algorithms used assume orthogonal periodic boundaries, which introduces additional limitations. To ensure proper treatment of all types of periodic boundary conditions, we therefore decided to use the boundary conditions to extend the system to neighboring unit cells prior to any other calculation. This extended trajectory is then aligned and used to calculate the densities from which the lipid-water interface is extracted. This will ensure that the interface and normal vector field will not suffer from any boundary effects in the central region of the extended simulation, corresponding to the unit cell of interest. We then use bolean flags to mark lipids for which tilts and splays should be calculated, which we usually take to correspond to the lipids from the central unit cell, but in general, can be any lipid set that is of interest (e.g., lipids on one leaflet only). This approach is both simple and efficient as it removes the necessity of treating periodic boundary conditions explicitely in the subsequent calculations.

### Code organisation, documentation, and examples

**Fig. 2:**
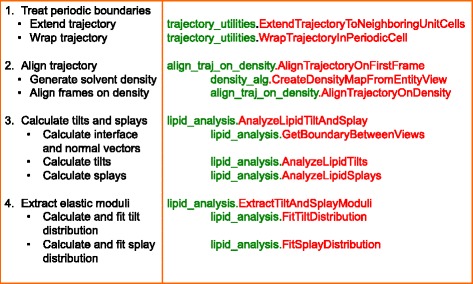
Typical workflow and associated functions. The entire procedure can be divided into four tasks: 1) Extending the trajectory using the periodic boundary conditions and wrapping it around the unit cell of interest (Schemes 1 and 2). This is done by using two functions of the *trajectory_utilities* module, *ExtendTrajectoryToNeighboringUnitCells* and *WrapTrajectoryInPeriodicCell*; 2) Aligning the trajectory using the *AlignTrajectoryOnFirstFrame* function (Scheme 3). This function first calculates the density of the solvent from the first frame of the MD trajectory using *CreateDensityMapFromEntityView* and then aligns the trajectory using *AlignTrajectoryOnDensity*; 3) Calculation of tilts and splays, using *AnalyzeLipidTiltAndSplay* (Scheme 4). This function first calculates the water lipid interface, and the field of normal vectors on the surface by calling the *GetBoundaryBetweenViews* function. Then it calculates the lipid tilts and splays using *AnalyzeLipidTilts* and *AnalyzeLipidSplays*; 4) Obtaining the elastic moduli by using the *ExrtactTiltAndSplayModuli* function which calls *FitTiltDistribution* and *FitSplayDistribution* (Scheme 5)

The main functions described here and used for computation of membrane elastic properties are contained in three modules (see Fig. 2 for a description of the standard workflow and associated functions). First the *trajectory_utilities* module contains general functions usefull when working with trajectories, such as wrapping a trajectory around a particular point, extending a trajectory to neighboring unit cells, and calculating the water lipid interface. The *align_traj_on_density* module contains the functions to align the frames of a trajectory on a reference frame using density maps, as described above. Finally the *lipid_analysis* module is the core of the method presented here, containing all functions for calculating lipid tilts and splays and extracting the membrane elastic moduli from their distributions.

The different functions are documented in the source code with *docstrings* and a full documentation can be generated using the *Sphinx* module or found online at http://njohner.github.io/ost_pymodules/. A full fledged example, including the MD trajectory, is available to illustrate the application of the method. Moreover, several code fragments for different scenarios provide scripts that can be easily adapted to the specific needs of different users. In these code fragments, the arguments passed to the different functions are explained and the parts that need to be modified for specific use cases are clearly marked. The source code and all other content is freely available on *GitHub* (https://github.com/njohner/ost_pymodules).

## Results and discussions

In the following, we will illustrate the use of the modules to perform the various steps needed to extract membrane elastic properties from MD trajectories, i.e.: extending and aligning trajectories, calculating the water-lipid interface, normal vector fields, and finally the lipid tilts and splays, and extracting membrane elastic properties from their distributions (see Fig. 2 for a complete workflow). Examples will be given either as excerpts from an interactive Ipython session (inputs preceded by **In [x]:** with **x** being the line number and outputs preceded by **Out [x]:**) or code fragments.

### Extending and aligning a trajectory

Let us start with a simple example of how to extend a trajectory so that it contains several unit cells and then wrap it around the unit cell of interest. As discussed in the *Implementation* section, this approach is used to treat periodic boundary conditions in later calculations. We assume here that we are working with a simple system containing a lipid bilayer spanning the *xy* plane (and periodic boundary conditions were used in the simulation).

**Scheme 1:**
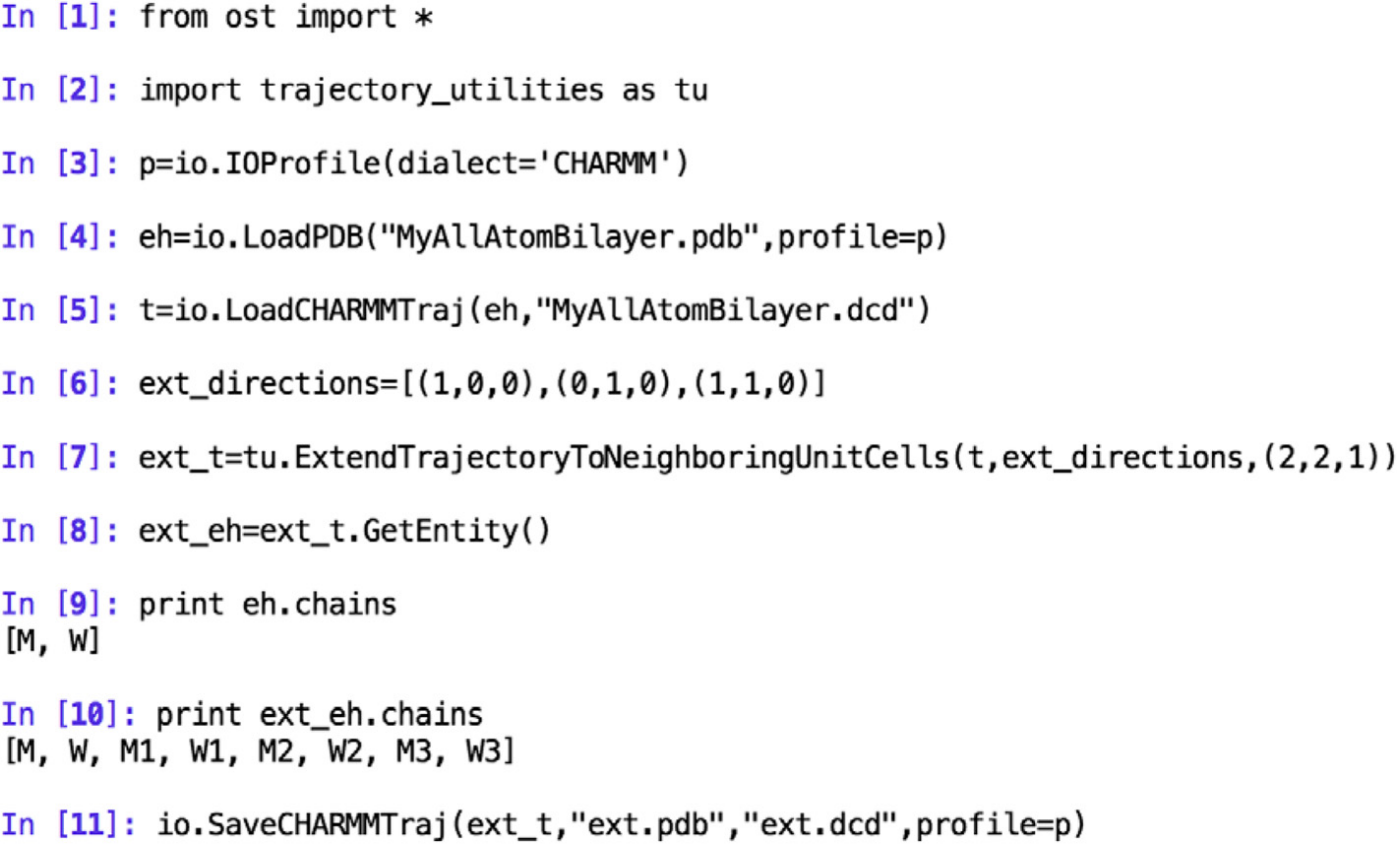
Loading and extending a trajectory

In this example we load a trajectory, extend it to include periodic images, and finally save the new trajectory to disk. Let us look at the code in Scheme 1 more closely. In the first line we import the *OpenStructure* computational framework, i.e. a set of modules among which are *io* (input-output module), *mol* (to work with structures) and *geom* (for working with geometrical objects such as vectors). In line 2 we import *trajectory_utilities*, one of the modules presented in this work. In lines 3-5 we load a structure (pdb file) and trajectory (dcd file). *eh* in line 4 is an *EntityHandle*, an object representing a molecular structure (chains, residues, atoms, bonds, etc.) and *t* in line 5 is a *CoordGroupHandle* containing the positions of the atoms in *eh* for every frame of the trajectory. More information about these objects can be found in the publications [22, 23] and documentation describing *OpenStructure*. Then, we extend the system by replicating and translating the unit cell according to the periodic boundary conditions (line 7). The directions of extension are specified by a list (here *ext_directions*) of triplets specifying combinations of the unit cell vectors used to generate the neighboring unit cells. In the example given above, we extend the system in the *xy* plane, i.e. we replicate the unit cell 3 times translating once along the first unit cell vector *(1,0,0)*, once along the second unit cell vector *(0,1,0)* and once along the sum of the first and second unit cell vectors *(1,1,0)*. The last argument passed on line 7 is a triplet of multiplication factors used to define the unit cell vectors of the extended trajectory. *ext_t* (line 7) is the extended trajectory (*CoordGroupHandle*) and in line 8 we retrieve the associated structure *ext_eh*. Lines 9 and 10 show that we initially had two chains in our structure, chain *M* correposonding to the membrane and chain *W* to the solvent. In the extended system we have 6 new chains (*M1*, *M2*, *M3*, *W1*, *W2* and *W3*), the membrane and solvent in the three new unit cells. In the last line of the example above, we save the extended trajectory (in a *pdb* and a *dcd* file).

**Scheme 2:**
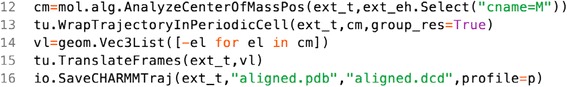
Aligning the trajectory for a planar bilayer

In the next code fragment (see Scheme 2), we wrap the trajectory around the unit cell of interest and then align the frames of the trajectory by superimposing the center of mass of the membrane of the central unit cell. Such an alignment is fast and works well for simple planar bilayers. Specifically, we first calculate the center of mass of the membrane of the unit cell of interest (chain with name *M* defined as “*cname=M*” in the query language of *OpenStructure* which is detailed in the online documentation) for each frame in the extended trajectory (line 12). This is done using the function *mol.alg.AnalyzeCenterOfMassPos* from *OpenStructure*. We then wrap each frame of the trajectory around the corresponding center of mass (line 13 in Scheme 2). The *group_res* flag ensures that residues will be kept whole when wrapping the positions. In lines 14 and 15, we translate each frame of the trajectory so that the center of mass of chain *M* is at the origin. Finally we save the aligned trajectory (last line of Scheme 2).

**Scheme 3:**
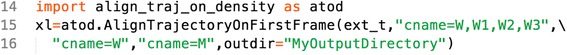
Aligning the trajectory for a lipidic phase of complex geometry

In the case of a lipid system with more complex (non-planar) geometry such as a simulation of an inverted-hexagonal phase, we would replace lines 14-16 of Scheme 2 with those shown in Scheme 3, which utilize the alignment algorithm described in the *Implementation* section. This is done by the *AlignTrajectoryOnFirstFrame* function of the *align_traj_on_density* module, as shown in Scheme 3. Specifically, this function will generate a density map of the solvent (*“cname=W,W1,W2,W3”*) from the first frame of the trajectory. Then it will align each frame by maximizing the overlap between the solvent of the central unit cell (*“cname=W”*) with that density, while minimizing the overlap of the lipids (*“cname=M”*) with it (see *Implementation* section for more information about the algorithm used). *Outdir* specifies the path to the directory where the output will be written (densities, aligned trajectory and list of transformations). The function returns a list of the transformations applied to each frame to align the trajectory.

This alignment method is very general but computationally time-consuming. It is therefore not advised to use this procedure for systems with simple planar geometries where the alignment can be performed using other approaches, as for example the one shown in Scheme 2.

### Calculating lipid tilts and splays

**Scheme 4:**
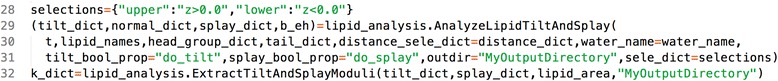
Calculating lipid tilts and splays

Once an extended and aligned trajectory is generated we can calculate the lipid tilts and splays and extract the membrane elastic properties from their distribution. The procedure is illustrated in Scheme 4. The implementation requires importing the *lipid_analysis* module, which is done in the first line of Scheme 4. In lines 2-5 we load the aligned (and extended) trajectory. This is followed by the definitions of the required arguments that will be passed to the *AnalyzeLipidTiltAndSplay* function, which calculates the tilts and splays from the trajectory. First we define the residues that constitute the solvent, in the given example these are *TIP3* water molecules. This will be used to select the solvent when determining the water-lipid interface. If there are additional types of molecules in the solvent, such as, for example, Na^+^ (*SOD*) and Cl^−^ (*CLA*) ions, simply use a coma separated list of their residue names: *water_name=”TIP3,SOD,CLA”*. This is followed by a list of all the lipid species (their residue name), in this example our bilayer only contains DPPC lipids (line 8 in Scheme 4). Next, for each lipid species present in the simulated system we must define the selections used as headgroup, tail and for distance calculations (see *Implementation* section and Fig. 1c for more details). This is done in dictionaries with one entry for each lipid species (lines 9-11 in Scheme 4). In the given example we use the center of mass of atoms *P* and *C2* as the position of the headgroup of *DPPC* lipids. Lastly, we are only interested in calculating lipid tilts and splays for the central unit cell, i.e. lipids in chain *M*. To this end, we attach 2 bolean flags *do_tilt* and *do_splay* to each *DPPC* molecule using the *SetBoolProp* method, setting them to *True* for lipids in chain *M* (lines 13-15, Scheme 4) and *False* for all the other lipids (lines 17-19, Scheme 4). Such custom properties are called *GenericProperties* in *OpenStructure* and can be attached to atoms, residues or chains.

**Scheme 5:**
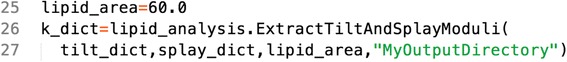
Extracting the tilt modulus and bending rigidity

**Scheme 6:**
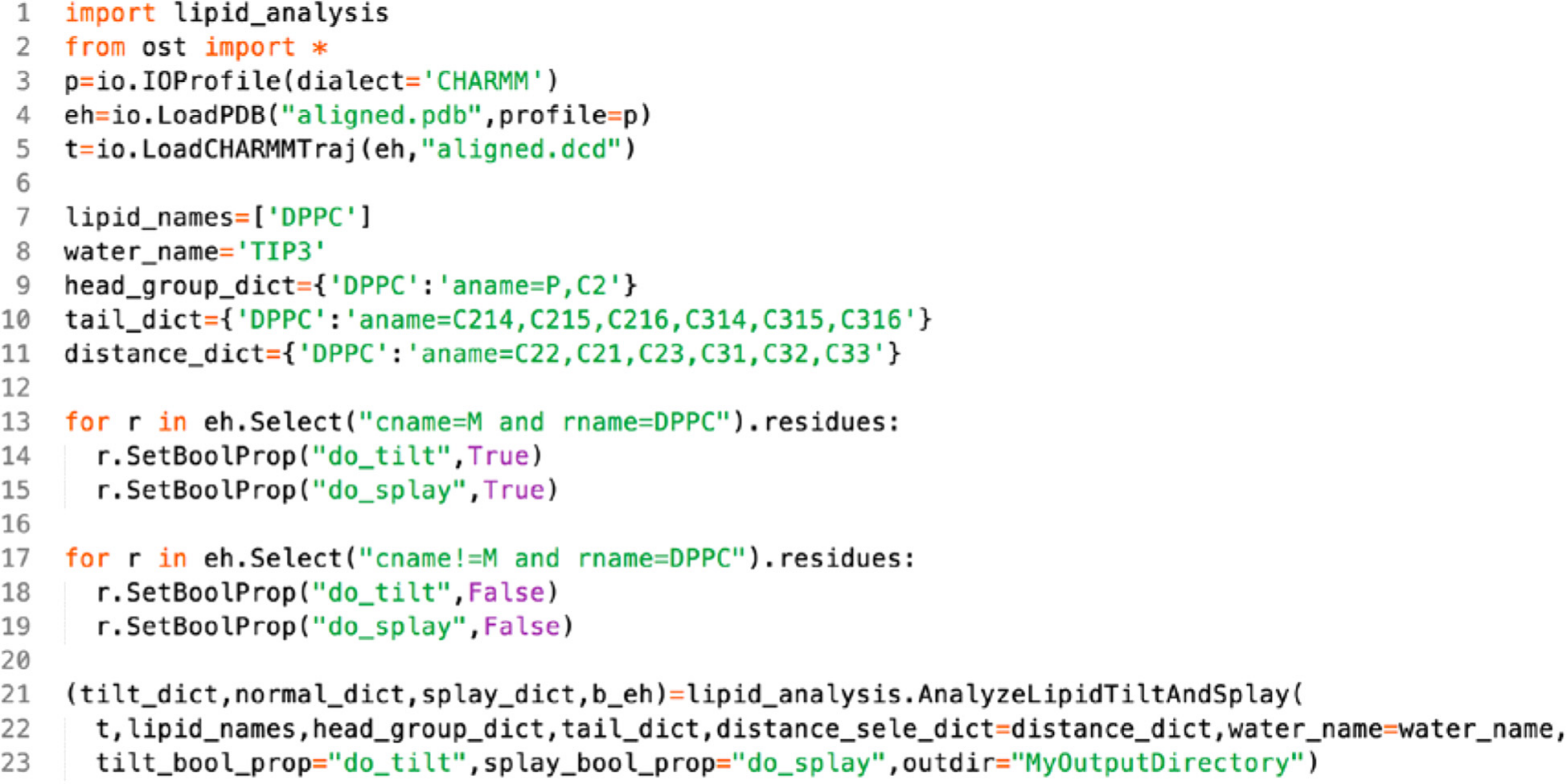
Calculate tilts and splays for each leaflet of a planar bilayer separately

We are now ready to calculate the lipid tilts and splays by calling the *AnalyzeLipidTiltAndSplay* function (lines 21-23, Scheme 4). The first argument passed is the trajectory, then the list of lipid species, followed by the dictionaries defining the headgroups and tails of the lipids. Then we pass the dictionary containing the selections used to calculate the distance between lipids, followed by the name of the molecules forming the solvent and the name of the bolean *GenericProperties* defining the lipids for which tilts and splays should be computed. Finally, we also indicate the path to the directory *MyOutputDirectory*, to which some output files will be written. Several other parameters, such as distance thresholds for splay calculations, can be set; refer to the documentation for a complete description of the arguments and their default values. The function returns; 1) a dictionary containing the lipid tilts; 2) a dictionary containing for each lipid and for each frame the normal vector of the closest point of the interface; 3) a dictionary containing the lipid splays; and 4) An *EntityHandle* representing the lipid-water interface. If an output directory is defined, the function will also write the water and lipid density maps, the interface and the normal vectors of the interface to that directory.

### Extracting the membrane elastic moduli

Once the lipid tilts and splays have been calculated, their distributions can be obtained and used to determine the elastic moduli by fitting eqs. 2 and 5 (see section *Theoretical Background*). The procedure can be performed using the *ExtractTiltAndSplayModuli* function (see Scheme 5). The function takes as inputs the tilt and splay dictionaries generated by the *AnalyzeLipidTiltAndSplay* function, as well as the area per lipid and an ouput directory. The area per lipid (taken here to be 60Å^2^ for DPPC as calculated from the MD trajectory) is required in the calculation of the bending rigidity (see eq. 5). The function will automatically calculate the distirbutions, determine the fitting ranges, extract the elastic moduli and estimate their uncertainties. It will also generate a series of figures, as well as text files containing the distributions and PMFs and the calculated elastic moduli, and save them to the output directory. It returns a dictionary containing the tilt and splay moduli and their uncertainties.

**Fig. 3:**
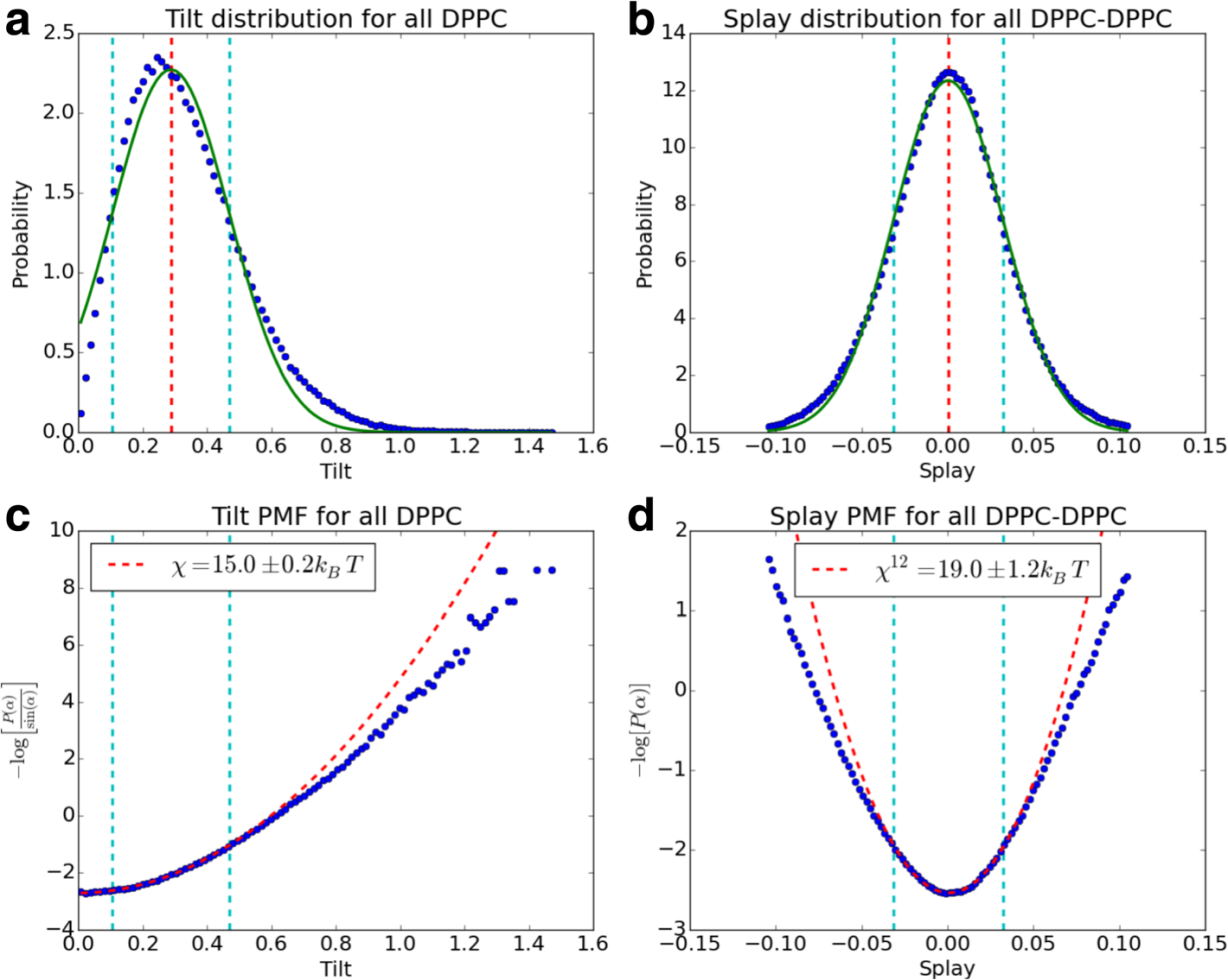
Tilt and splay distributions and fits. Example of the plots automatically generated by the *ExtractTiltAndSplayModuli* function for the case of a simple DPPC planar bilayer. (**a**) The distribution of tilts *P (θ)* with the fitted gaussian *G*(*μ*, *σ*) in green. The mean of the distribution *μ* is shown as a red dashed line while the fitting interval [*μ* − *σ*, *μ* + *σ*] is shown as blue dashed lines. (**b**) Same as (a) but for lipid splays. (**c**) The PMF for lipid tilts with the corresponding quadratic fit (eq. 2) shown as a dashed red line. (**d**) The PMF for lipid splays with the corresponding fit (eq. 5) shown as dashed red line. Extracted elastic constants are shown in the legends

The details of the procedure are as follows: 1) We compute the distributions of tilts *P*(*θ*) and splays *P*(*S*_*i*_) and fit a gaussian to each of them to determine their mean *μ* and standard deviation *σ*. These distributions are plotted and saved to the ouput directory (Fig. 3a and b). 2) We calculate the PMFs for the tilts and splays (eqs. 2 and 5) and fit them with a quadratic function. Five different fitting ranges are used [*μ* − *cσ*, *μ* + *cσ*]; *c* ∈ {1, 1.25, 1.5, 1.75, 2}. The modulus is taken as the one obtained form the fit with *c* = 1 and the uncertainty as the standard deviation of the 5 calculated moduli. Again plots of the PMFs and their fit are generated (Fig. 3c and d). All reported moduli are per monolayer and are given in units of *k*_*B*_*T*.

### Lipid mixtures and assymetric membranes

In the case of membranes composed of a mixture of lipids, we calculate tilt moduli separately for each lipid species and obtain individual splay moduli for each pair of lipid species. The monolayer tilt and splay moduli are then obtained using:

8 $$ \frac{1}{K_C}=\frac{1}{\phi_{Tot}}{\displaystyle \sum_{i,j}}\frac{\phi_{ij}}{\chi_{ij}^{12}} $$

9 $$ \frac{1}{\kappa_t^l}=\frac{1}{N_L}{\displaystyle \sum_i}\frac{N_{Li}}{\chi_i} $$

where *χ*_*ij*_^12^ is the splay modulus for the pair of lipid species *i* and *j* and *χ*_*i*_ the lipid tilt modulus for lipid species *i. N*_*Li*_ is the number of lipids of species *i* and *N*_*L*_ the total number of lipids. *ϕ*_*ij*_ is the number of splays calculated in the MD trajectory for pairs of lipids of species *i* and *j,* and *ϕ*_*Tot*_ the total number of lipid splays. Note that, in the case of lipid mixtures, the area per lipid, which enters the calculation of the splay moduli for each pair of lipid species (eqs. 4 and 5), is approximated as being the same for all lipid species and can be obtained (for a planar bilayer) as the area of the unit simulation cell in the membrane plane divided by the total number of lipids per leaflet (for example using the *AnalyzeAreaPerLipid* function of the *lipid_analysis* module). As we have demonstrated previously [21, 26], this formulation has yielded bending rigidity values for many different binary and tertiary mixtures in good agreement with experimental data. Using different areas for each lipid species, in principle, is possible within the overall formalism, but would require reweighting the contributions of each pair of lipids in eq. 8. We leave any such modification for future work.

For asymetric bilayers (with different compositions in the two leaflets), the elastic moduli can be calculated seperately for each leaflet. This is done by passing as optional argument to *AnalyzeLipidTiltAndSplay* a dictionary containing selection strings that are used to divide the system into parts that are treated independently. Scheme 6 shows an example of how to split the calculation for upper and lower leaflets for a planar lipid bilayer with its center of mass at the origin (i.e., z=0). Elastic moduli would then be computed separately and plots and files would be generated for each of the leaflets in that case.

## Conclusions

The need for accurate determination of membrane elastic properties for physiologically realistic lipidic assemblies is growing, as such information is essential for our understanding of the lipids’ role in a wide range of cellular processes [33] and as we continue to uncover the immense variety of lipids in cells [34, 35]. Extensively validated against experimental results [21, 32], the method we developped is, to the best of our knowledge, the only available computational framework for treating multi-component lipid systems and/or curved lipidic assemblies. The implementation as python modules presented here offers great flexibility in the application of the method to a wide variety of systems. The modules allow the calculation of membrane elastic properties from molecular dynamics simulations of lipid assemblies of arbitrary shape and composition. It also allows to work with any type of lipids and force-fields because the user manually defines the various selections, such as solvent, lipids, lipid head groups, etc. The modules also implement solutions to deal with the specific problems arising when working with curved membranes and complex lipidic phases, notably aligning the trajectories and calculating the water-lipid interface and field of normal vectors. Overall the modules provide complete tools to extract the tilt and bending elastic material moduli from MD simulations of any type of lipidic assemblies and, as such, should help the community in our effort to characterize and understand the physico-chemical properties of complex multicomonent lipid assemblies.

## Availability and requirements

**Project name:** ost_pymodules

**Project home page:**https://github.com/njohner/ost_pymodules

**Operating system(s):** Platform independent

**Programming language:** Python

**Other requirements:** OpenStructure, python 2.7, numpy, scipy, matplotlib

**License:** GNU Lesser General Public License

**Any restrictions to use by non-academics:** None

## Abbreviations

MD: Molecular dynamics
PMF: Potential of mean force
*EntityHandle*: Object used to represent a molecular structure
*CoordGroupHandle*: Object used to store the positions of the atoms form a trajectory
