# Erratum to: Implementation of a methodology for determining elastic properties of lipid assemblies from molecular dynamics simulations

**DOI:** 10.1186/s12859-016-1091-9

**Published:** 2016-06-14

**Authors:** Niklaus Johner, Daniel Harries, George Khelashvili

**Affiliations:** Swiss Institute of Bioinformatics, Klingelbergstrasse 50/70, Basel, Switzerland; Institute of Chemistry and the Fritz Haber Research Center, The Hebrew University, Jerusalem, 91904 Israel; Department of Physiology and Biophysics, Weill Medical College of Cornell University, New York, NY 10065 USA

Unfortunately, the original version of this article [1] contained an error. The incorrect version of Scheme 4 was used and Scheme 4 and 6 were also accidentally interchanged during processing. The correct schema and labelling is presented below.
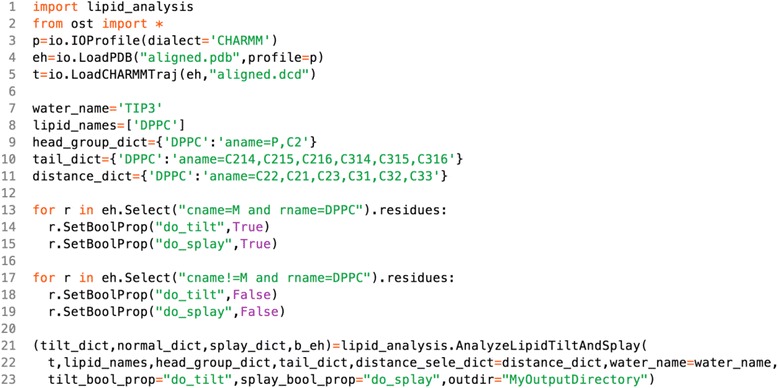


Scheme 4. Calculating lipid tilts and splays
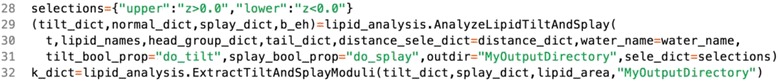


Scheme 6. Calculate tilts and splays for each leaflet of a planar bilayer separately
